# Multi-sample SPIM image acquisition, processing and analysis of vascular growth in zebrafish

**DOI:** 10.1242/dev.173757

**Published:** 2019-03-21

**Authors:** Stephan Daetwyler, Ulrik Günther, Carl D. Modes, Kyle Harrington, Jan Huisken

**Affiliations:** 1 Max Planck Institute of Molecular Cell Biology and Genetics, 01307 Dresden, Germany; 2 Department of Cell Biology, UT Southwestern Medical Center, Dallas, TX 75390, USA; 3 Center for Systems Biology Dresden, 01307 Dresden, Germany; 4 Chair of Scientific Computing for Systems Biology, Faculty of Computer Science, TU Dresden, 01069 Dresden, Germany; 5 Virtual Technology and Design, University of Idaho, Moscow, ID 83844, USA; 6 Morgridge Institute for Research, Madison, WI 53715, USA; 7 Department of Integrative Biology, University of Wisconsin, Madison, WI 53706, USA

**Keywords:** Multi-sample imaging, Light sheet microscopy, Zebrafish, SPIM, Vasculature, Segmentation, Growth models

## Abstract

To quantitatively understand biological processes that occur over many hours or days, it is desirable to image multiple samples simultaneously, and automatically process and analyse the resulting datasets. Here, we present a complete multi-sample preparation, imaging, processing and analysis workflow to determine the development of the vascular volume in zebrafish. Up to five live embryos were mounted and imaged simultaneously over several days using selective plane illumination microscopy (SPIM). The resulting large imagery dataset of several terabytes was processed in an automated manner on a high-performance computer cluster and segmented using a novel segmentation approach that uses images of red blood cells as training data. This analysis yielded a precise quantification of growth characteristics of the whole vascular network, head vasculature and tail vasculature over development. Our multi-sample platform demonstrates effective upgrades to conventional single-sample imaging platforms and paves the way for diverse quantitative long-term imaging studies.

## Abstract (precis)

**Summary:** A novel dedicated multi-sample light sheet imaging, processing and analysis platform is presented, and its value in diverse quantitative long-term imaging studies, such as vascular growth in zebrafish, is demonstrated.

## INTRODUCTION

The cardiovascular system is among the earliest functional organs to be formed during vertebrate development. From a few individual mesodermal precursor cells, a complex network of vessels forms through a variety of morphogenetic processes (Ellertsdóttir et al., 2010; Gore et al., 2012). Although many molecules involved in its formation have been identified (Adams and Alitalo, 2007; Gore et al., 2012; Hogan and Schulte-Merker, 2017), much remains unknown about how this intricate network comes into shape at the whole embryo level. A particularly exciting unanswered question is how the volume of the vascular system changes over development. Vascular volume is a strong proxy for overall vascular system size and consequently its development provides insight into fundamental aspects of tissue development and whole-embryo morphogenesis. Here, we show how a combination of dedicated multi-sample preparation, comprehensive imaging and data processing, a novel segmentation approach, and growth data analysis provide a precise and quantitative characterization of embryonic vascular volume development.

To understand vascular formation at the whole-embryo level, the zebrafish has become a very valuable tool with many available vascular transgenic lines (Chávez et al., 2016). Compared with other vertebrate model organisms such as mice, the development of zebrafish is fast and takes place outside of the mother, and the optical translucency of zebrafish embryos provides an ideal setting for long-term *in vivo* time-lapse imaging experiments (Kimmel et al., 1995).

For long-term live imaging of zebrafish embryos and larvae, light sheet microscopy (Huisken et al., 2004; Keller, 2013) has become the method of choice due to its illumination and detection scheme, which provides minimal photo-bleaching and phototoxicity (Daetwyler and Huisken, 2016; Icha et al., 2017; Power and Huisken, 2017). Moreover, light-sheet microscopy offers sample rotation in the microscope for multi-view imaging and tiling for full-embryo coverage (Weber and Huisken, 2012), which is a necessity for imaging the entire vascular system. In addition, imaging zebrafish embryos over several days requires a sample embedding technique that provides mechanical constraints to ensure proper sample orientation but at the same time does not limit oxygen access or restrict growth. For single samples, an embedding method using fluorinated ethylene propylene (FEP) tubes (Kaufmann et al., 2012) has been widely accepted. However, to quantitatively analyse the observed processes, several samples need to be imaged. Ideally, these are imaged in one experiment simultaneously, which is especially important and efficient if the experiment takes several days to complete. Therefore, multi-sample imaging is highly desirable for long-term imaging studies.

The number of samples during imaging can be increased either by delivering samples with flow (Gualda et al., 2015; Regmi et al., 2013; Regmi et al., 2014; Wu et al., 2013) or by embedding multiple samples at the same time (de Luis Balaguer et al., 2016; Schmid et al., 2013). Delivering samples by flow does not offer the precise control of sample orientation needed for optimal image quality and comparison of different time points. Therefore, multi-sample embedding solutions are more promising for long-term imaging studies. However, existing multi-sample embedding solutions are currently only suitable for embryos still in their protective envelope, the chorion (Schmid et al., 2013), but not for growing zebrafish larvae. Sample holders for several plants have been designed to allow plant growth in near physiological condition (de Luis Balaguer et al., 2016) but do not provide sample rotation during imaging to access the entire sample. Therefore, the challenge is to develop a multi-sample embedding technique that allows for multi-view imaging.

A further complication arises when imaging several embryos simultaneously, as data handling becomes more challenging, with datasets easily exceeding several terabytes in size. Furthermore, such datasets comprise many acquisition volumes, angles and time points over multiple samples. It is consequently not possible to load an entire experiment into computer memory to inspect the data and apply conventional data processing and visualization workflows. Therefore, custom data processing tools are needed to automatically generate 3D stitched datasets for further analysis, and to visualize the acquired data for easy inspection.

Next, for an accurate description of vascular volume changes, visual inspections and qualitative analysis are not sufficient. Instead, quantitative measurements based on vascular segmentation are required. Most segmentation approaches in developmental biology focus on analysing nuclei and cytoplasmic content (Amat et al., 2014), but segmentation of the vasculature is more challenging because of the variety of vessel sizes, intensity changes over vessel walls and, most importantly, due to the hollow-tube structure of vessels. Therefore, no reliable segmentation of endothelial signal over long time periods has been established yet. To help with segmentation, micro-angiography (Isogai et al., 2001) is often used, in which a fluorescent dye is injected into the vasculature. However, at early developmental stages, the vasculature is not completely closed and dye rapidly leaks into the surrounding tissue, rendering microangiography not applicable for long-term developmental studies. A novel strategy of vascular segmentation is therefore required to extract quantitative growth measurements of the whole vasculature.

To interpret the quantitative measurements resulting from the complete multi-sample imaging and processing platform, there are many established mathematical growth models available (Hernandez-Llamas and Ratkowsky, 2004; Tsoularis and Wallace, 2002), such as the Gompertz model (Gompertz, 1825), logistic growth model (Verhulst, 1838), the Weibull model (Ratkowsky, 1983) or Richards’ model (Richards, 1959; Tjørve and Tjørve, 2010). However, none of these models is capable of sufficiently describing our data. We therefore suggest a new class of growth models based on logarithmic rescaling of time to describe embryonic vascular development.

## RESULTS

### Multi-sample preparation for long-term time-lapse imaging

**Fig. 1.**
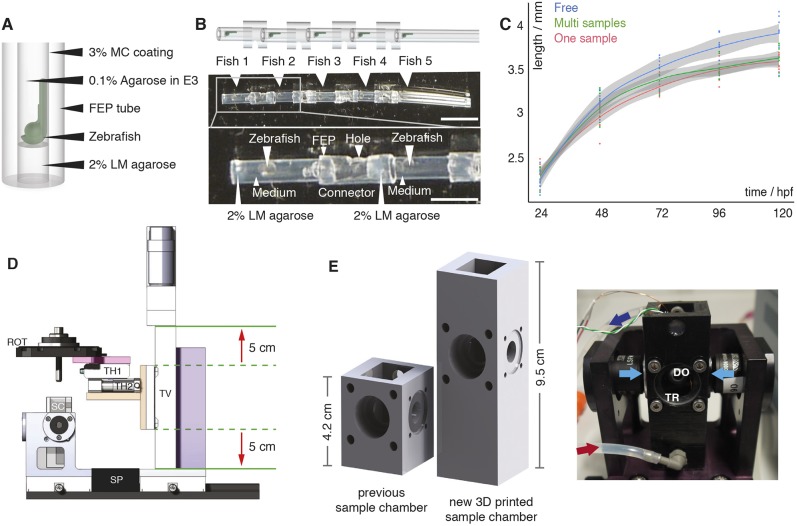
**Multi-sample embedding and necessary microscope modifications.** (A) Schematic of single zebrafish embryo embedding in a fluorinated polypropylene (FEP) tube using 2% low melting point (LM) agarose as a plug, 0.1% agarose in E3 as the medium and 3% methylcellulose (MC) for coating the FEP tube, as described previously (Kaufmann et al., 2012). (B) Schematic (top) and images (middle) of five mounted zebrafish (white arrowheads) in FEP tube pieces assembled using FEP connectors. The bottom image shows details of the embedding: zebrafish mounted in the FEP tubes rested on a 2% LM agarose plug and were embedded in E3 medium containing 0.1% agarose. FEP tubes containing a single zebrafish embryo were attached with FEP connectors containing a hole. Scale bar: 1 cm (top image); 4 mm (bottom image). (C) Growth curve of freely swimming fish (blue, *n*=8), individually embedded samples (red, *n*=10) and samples embedded in the multi-sample tube (green, *n*=9) with the 0.95% confidence interval of Loess interpolation in grey. (D) The new translational stage design of the multi-sample imaging platform ensured a vertical travel range of 10 cm (red arrows) and was built with custom-made parts, e.g. an adapter (pink) between the rotational stage (ROT) and the translational stage platform, an adapter (orange) between the horizontal translational stages (TH1 and TH2) and the vertical translational stage (TV), and a stage mount (purple). SP, spacer; SC, sample chamber. (E) Comparison of a traditional one-sample SPIM chamber with the new 3D-printed sample chamber and a picture of the new sample chamber connected to a perfusion system with inflow (red arrow) and outflow (dark blue arrow), the two illumination objectives for dual-sided illumination (light-blue arrows), a window for transmission (TR), and a detection objective (DO) in the back.

Successful vascular imaging relies on immobilization of the sample and mechanically constraining it to the field of view through embedding. In contrast to the widely established zebrafish embedding protocol for light sheet microscopy (Kaufmann et al., 2012), we abstained from using tricaine. Instead, to immobilize the embryos, we injected them with α-bungarotoxin RNA (Swinburne et al., 2015) at the one-cell stage. To embed several samples for simultaneous long-term imaging, we adapted the established protocol of single-embryo embedding. We first embedded individual zebrafish embryos in 0.1% agarose inside of FEP tubes for long-term imaging as described previously (Kaufmann et al., 2012) (Fig. 1A, Figs S1 and S2). These FEP tubes were cut to a length of about 8 mm and several of them were attached with other larger FEP tubes as connectors (Fig. 1B). Holes in these connectors ensured exchange of oxygen and liquids during imaging. This embedding technique was readily available, flexible and provided stable embedding of up to five fish for simultaneous, long-term imaging in one tube assembly with a length of about 6 cm (Fig. S3). A detailed step-by-step protocol for multi-sample embedding can be found in the supplementary Materials and Methods. We also include suggestions for how to solve potential embedding problems (Table S1) and a detailed list of required embedding materials (Table S2).

To ensure that the multi-sample embedding did not compromise growth, we measured the overall body length of freely swimming zebrafish, single-sample and multiple-sample preparations over time, and compared their growth curves (Fig. 1C). Until 24 h after embedding [48 h post fertilization (hpf)], no growth difference was detected (ANOVA analysis, *P*-value 0.1721). This changed at 72 hpf (ANOVA analysis, *P*-value 0.0013), when the embedded fish were 5% smaller than the freely swimming fish after 2 days of embedding. However, the data provided no evidence that there was a growth impairment of the multi-sample embedded fish compared with the established one-sample embedding technique (two-sided *t*-test between one-sample and multi-sample embedding: *P*=0.06 at 72 hpf, 0.93 at 96 hpf and 0.98 at 120 hpf). Moreover, no additional growth defects such as oedemas were detected by visual inspection (Fig. S4).

### Hardware adaptations for multi-sample imaging

The new 6 cm long tube assembly containing the embedded zebrafish embryos did not fit on our custom and other commercially available light-sheet microscopes. Therefore, we decided to upgrade an existing custom three-lens mSPIM microscope (Huisken and Stainier, 2007) that has been successfully used in long-term imaging studies (Lenard et al., 2015). The existing mSPIM microscope was equipped with two illumination arms and one orthogonal detection arm. Furthermore, its rotational stage allowed us to rotate zebrafish embryos and image them from optimal angle(s) for better penetration and coverage. To upgrade this custom system, we designed a new translational stage system (Fig. 1D), with larger travel ranges to move every one of the five samples into the microscope's field of view. Furthermore, the depth and height of the sample chamber (Fig. 1E) was increased to provide enough space for the longer tube assembly. The taller chamber was 3D printed (Fig. 1E), making the chamber a cost-effective and easily adaptable unit. We incorporated in- and outlets for a perfusion system into the sample chamber to allow for temperature control of the medium and sample.

### Multi-sample imaging of several zebrafish embryos

To study the development of the vascular volume, we simultaneously imaged five zebrafish embryos expressing a green fluorescent vascular endothelial marker, *Tg(kdrl:EGFP)* (Jin et al., 2005), and a red blood cell marker, *Tg(GATA1a:dsRed)* (Traver et al., 2003). The green fluorescent marker labelled the vessel walls and thus the outlines of the vascular system. The red blood cell marker labelled erythrocytes and thus provided information about the inside of the vasculature. To achieve optimal coverage of the entire vascular system, we chose three optimal angles for imaging (Fig. S5): to capture a good view on the head vasculature, we imaged the embryos with dual-sided illumination with the detection oriented dorsally; to achieve a good whole-embryo view, we imaged the zebrafish from two opposite angles ±60° rotated in the sagittal plane with single-sided illumination.

For acquisition, we used EMCCD cameras with a chip size of 960×960 pixels corresponding to a small 1.097×1.097 mm field of view when using our 10× lens. As the embryos grew to a size of about 3.5 to 4 mm at 5 dpf, several acquisition volumes were required to reconstruct the whole embryos. In total, together with an overlap of about 30% for reliable data stitching, five acquisition volumes were needed to image one whole embryo from one angle. Therefore, for imaging five embryos from three angles, 75 acquisition volumes were required. To capture the total 3D volume, we imaged every acquisition volume with at least 200 *z*-planes separated by 4 µm.

To image the development of the vascular volume over time, we began imaging at around 17 hpf when the cells started to fuse to larger vessels by *in situ* aggregation of angioblasts (Ellertsdóttir et al., 2010), and imaged the entire developing vasculature every 20 min over a time period of at least 3 days. With these settings (75 acquisition volumes, dual-colour 16-bit images, 200 planes/acquisition volume, time step of 20 min), the corresponding data generation rate amounted to ∼3.5 TB/day.

### Microscope software adaptations for multi-sample imaging

The high data rate from our multi-sample imaging SPIM instrument posed a considerable challenge, as hard-drives available for the microscope had a total capacity of only 6 TB, making a 3-day time-lapse experiment impossible. We therefore decided to copy the accumulated data from every time point to a large, central storage unit in between acquisitions (see Materials and Methods, Fig. S6). This approach also eliminated the usual time-consuming data transfer at the end of an experiment, which prevents the immediate start of the following experiment. Copying data during an experiment thus also increased the overall throughput of the microscope.

We further implemented an automated mosaic generation tool, because manually configuring a high number of individual acquisition volumes for each experiment is very time consuming and error prone. Given a starting position, e.g. the head of the fish, the other acquisition volumes were automatically determined with a 30% overlap, ensuring reliable stitching during data processing. The overall number of acquisition volumes was selected based on the expected growth of the zebrafish over the course of the time-lapse at the start of the experiment.

### Dedicated processing pipeline

Multi-sample acquisition over 3 days as described above resulted in large datasets of over 10 TB with a convoluted data structure comprising many acquisition volumes, several angles and fish, and two channels. Using custom modular ImageJ/Fiji plug-ins (Schindelin et al., 2012), we transformed the raw data into a dataset that contained one stitched 3D stack per timepoint, channel, angle and fish (Figs S7, S8). Furthermore, to visually check the data quality, we generated maximum intensity projections that also enabled a qualitative description of the formation of the vasculature (Movies 1 and 2). We adapted the plug-ins for use on a high-performance cluster for fast processing (supplementary Materials and Methods). Using the microscope stage parameters, we also automated all processing steps so that in the end only the folder of the experiment had to be given by the user. The code for all processing plug-ins is freely available at github.com/DaetwylerStephan/multi_sample_SPIM.

### Segmentation of the vascular data

**Fig. 2.**
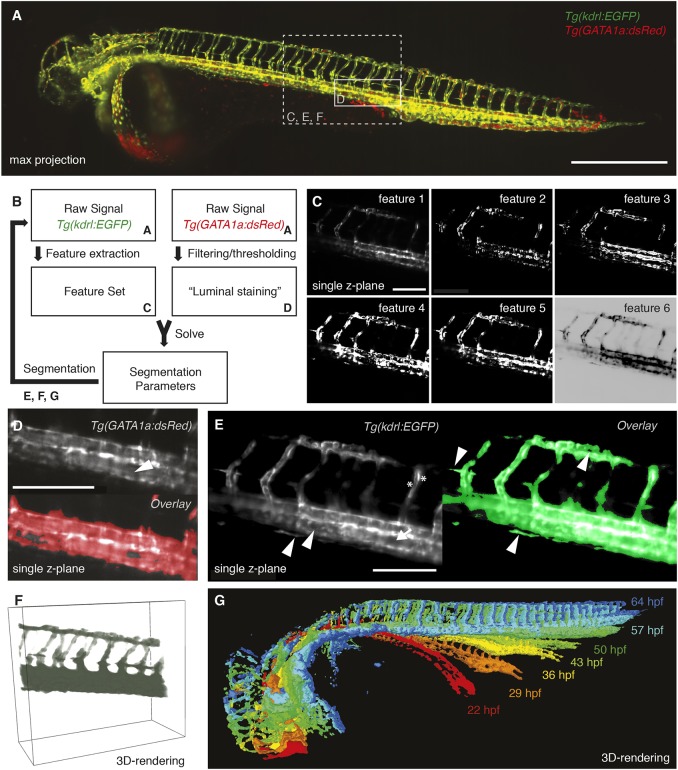
**Segmentation of the vascular data.** (A) Maximum intensity projection of a zebrafish expressing the endothelial marker *Tg(kdrl:EGFP)* in green and the red blood cell marker *Tg(GATA1a:dsRED)* in red with boxes depicting selected regions shown in C-F. Scale bar: 500 µm. (B) Schematic of the segmentation process with references to the corresponding figure panels (A,C-G). For successful segmentation, the imagery data of two channels was required: the raw data of the vascular marker *Tg(kdrl:EGFP)* and of the red blood cell marker *Tg(GATA1a:dsRed)* (A). (C) From the vascular marker, a feature set (C) was extracted. By filtering and thresholding the images of the red blood cell marker, a proxy for a luminal staining was established. The weights for the different features (segmentation parameters) were then calculated by using the luminal proxy as ground truth in a machine learning-based approach. With the obtained feature weights, the segmentation of the vascular channel was achieved. Features extracted from the endothelial marker *Tg(kdrl:EGFP)* included the raw signal (feature 1), gradient x (feature 2), gradient y (feature 3), gradient z (feature 4), total gradient (feature 5) and the inverse gradient weighted raw image (feature 6). Scale bar: 150 µm. (D) Single plane of a 3D stack of the red blood cell marker *Tg(GATA1a:dsRED)* (top). As the red blood cells circulate in the vasculature, the interior of vessels was also filled with fluorescent signal (white arrowhead). Therefore, filtering and thresholding of the red blood cell marker raw signal (top) provided a ground truth (bottom, red) of signal inside the vasculature from which the segmentation parameters could be calculated. Scale bar: 150 µm. (E) Single plane of a 3D stack of the endothelial marker (left) highlighting the challenges of vascular segmentation: hollow tubes (arrow), intensity differences (asterisks) and small vessels next to a large vessel (arrowheads). Using our segmentation approach (right), even fine structures of the vasculature were segmented correctly (arrowheads). Scale bar: 150 µm. (F) 3D rendering of the selected region with the segmentation in green. (G) 3D rendering of the segmentations at different time points of development.

To quantitatively measure the vascular volume changes over time, a segmentation of the vasculature (Fig. 2A) was required. To provide an effective segmentation, we designed a novel approach (Fig. 2B) by complementing the signal of the endothelial marker with the signal from the red blood cell marker *Tg(gata1a:DsRed).* We included the second marker because red blood cells circulate inside the vasculature and are therefore inherent proxies for a luminal marker. This provided ideal training data for a machine learning-based approach of vascular segmentation.

To establish the training set for solving the segmentation, we determined candidate locations for each individual 3D stack. Those locations were selected by taking the conjunction of the vasculature channels (Fig. 2C) and the thresholded red blood cell (Fig. 2D). Sample points were chosen by randomly sampling candidate coordinates and collecting the first *N* positive points and first *N* negative points without replacement (*N*=5000). The sample points were used as a target for the parameter fitting procedure.

For every sample point, the features from the vascular marker were extracted using first-order moments calculated with ImgLib2 (Pietzsch et al., 2012). The features encoded the original image (Fig. 2C, feature 1), X, Y and Z gradients (Fig. 2C, feature 2-4) of the vasculature channel, the total gradient magnitude (Fig. 2C, feature 5), and an inverse-gradient weighted version of the original image (Fig. 2C, feature 6). The feature vectors for all sample points were then composed as a matrix and a target vector was generated, representing the positive/negative labels of training points as 1 and 0, respectively. Singular value decomposition was then used to solve for the segmentation parameters, i.e. a set of feature weights that maximized predictions of the target vector value.

The feature weights obtained from solving the linear fitting procedure were then used to compute an image of segmentation scores. Images were segmented by first using the fitted feature weights to compute a linear combination of the feature maps. The resulting image encoded the segmentation score for each voxel. The segmentation score image was then thresholded using the Triangle algorithm (Zack et al., 1977), resulting in a binary labelling of the image. A morphological erosion followed by a dilation filtered out single-pixel fragments.

With this novel segmentation approach in hand, the various challenges of vessel segmentation posed by vessel geometry and characteristics of the fluorescent marker (Fig. 2E) were overcome. The segmentation approach resulted in a segmentation that was capable of capturing even fine morphological details of vascular structures (Fig. 2E,F, Fig. S9).

As the segmentation had to cope with terabytes of data from the many unlabelled 3D stacks, we implemented our efficient and fully automated image segmentation pipeline in FunImageJ (Harrington et al., 2018). FunImageJ was used with a standard distribution of Fiji (Schindelin et al., 2012), and as a standalone program on a high-performance computing cluster to enable parallel processing of whole datasets. The resulting segmentations were then used to quantify growth of the vascular system over several days (Fig. 2G).

### Vascular growth curve rates

**Fig. 3.**
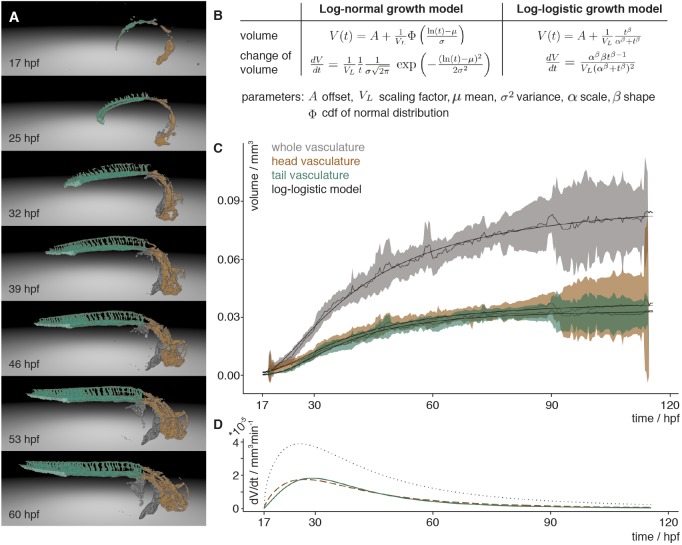
**Vascular volume growth characteristics of zebrafish.** (A) Segmentation of the vasculature at seven different time points labelled with the annotation of the head (orange) and tail (turquoise) with its caudal vein plexus (light turquoise) and the rest of the segmented vasculature (grey). (B) Equations of the scaled cumulative log-logistic and log-normal growth models describing the volume *V*(*t*) and change of volume *dV*/*dt* over time *t* with offset *A*, scaling factor *V_L_*, mean *µ* and standard deviation *σ* of log-normal distribution and cumulative distribution function (cdf) Φ of the standard normal distribution, and log-logistic scale parameter *α* and shape parameter *β*. (C) Experimental measurements of the volume over time of the whole vasculature (grey), and the head (brown) and tail (turquoise) vasculature. The mean of the measurements is depicted with a solid line and the 95% confidence interval (t-statistics, *n*=7) as a ribbon in the corresponding colour. The black line depicts the approximation of the volume calculated using the scaled cumulative log-logistic growth model. The corresponding panel for the scaled cumulative log-normal model is in Fig. S10A. (D) The volume growth rate of the whole vasculature (grey, dotted), and head (brown, dashed) and tail (turquoise) vasculature was calculated by inserting the parameters obtained from the approximation into the change of volume equation of the scaled log-logistic growth model. The corresponding panel for the scaled log-normal model is in Fig. S10B.

With the segmentation in hand (Fig. 3A), quantifying the overall growth of the vascular system was a straightforward process of computing the number of segmented voxels in isotropic 3D stacks. To temporally align the volume measurements of different fish, we selected the anastomoses of the left and right primordial hindbrain channel (PHBC) as reference points.

The resulting quantitative data revealed that the growth of the vascular volume could be well described by a group of models relying on logarithmic rescaling of time, including the scaled cumulative log-logistic (Fig. 3B) and the scaled cumulative log-normal growth models (Fig. S10). To account for a limited time window of observation, a scaling parameter and an offset were introduced in both models. The offset *A* described the lower asymptote, i.e. the vascular volume already formed at the start of an imaging experiment. The scaling factor *V_L_* described the upper asymptote, i.e. the maximum volume reached during the observed development. In addition, for the scaled cumulative log-logistic model, the scale parameter α and the shape parameter β were required to describe vascular volume growth (Fig. 3C). For the scaled cumulative log-normal growth model, two parameters µ (mean) and σ (standard deviation), which define the underlying log-normal function, were required. Consequently, in total only four parameters were required to model the development of the vascular volume over time. We used non-linear minimization to obtain the fitting parameters for both models (Table 1).

**Table 1.**
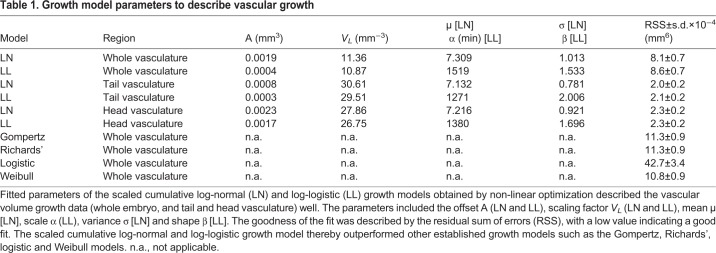
**Growth model parameters to describe vascular growth**

We compared the scaled cumulative log-normal and log-logistic growth model with other established growth models by calculating the residual sum of squares (RSS). A small RSS indicates a good fit to the data. The comparison of the RSS values revealed that the scaled cumulative log-normal and log-logistic growth models of the whole vasculature volume growth exceeded other established growth models, such as the Gompertz model (Gompertz, 1825), logistic growth (Verhulst, 1838), the Weibull model (Ratkowsky, 1983) or Richards’ model (Richards, 1959; Tjørve and Tjørve, 2010) (Table 1, Fig. S11). Moreover, visual inspection of the residuals of the scaled cumulative log-normal and log-logistic growth model also indicated that those models fit best (Fig. S11).

To understand whether the overall growth characteristics of the whole vasculature were reflected in the growth of its subnetworks, such as the head or tail vasculature, we established a manually curated annotation of the vasculature (Movie 3). The volume changes of the differently annotated regions were determined by computing the number of segmented voxels with the corresponding annotation label in isotropic 3D stacks. The analysis of vascular volume development of the head and tail vasculature (Fig. 3C) revealed that they were also well described by the scaled cumulative log-logistic and log-normal models. The fitting parameters for both models were obtained by linear minimization and revealed a good fit (Table 1).

We inserted the above obtained parameters into the scaled log-logistic function on which the scaled cumulative log-logistic model was based (Fig. 3B). The scaled log-logistic function was the derivative of the scaled cumulative log-logistic model and thereby revealed the growth rate of the vascular volume (Fig. 3D). The maximal growth rate obtained was around 26 hpf for the whole vasculature, 27 hpf for the head and 29 hpf for the tail vasculature. Before this, the growth rate rapidly increased then, after reaching the maximum, the growth rate slowly decreased. Inserting the parameters of the scaled cumulative log-normal model into its derivative revealed the same values for whole vasculature and head vasculature, and 28 hpf for the tail vasculature (Fig. S10B).

## DISCUSSION

We have presented a dedicated and complete workflow of multi-sample preparation, multi-sample imaging, data processing and quantitative analysis of the vascular volume. Several key innovations in all those disciplines were necessary: we introduced a new multi-sample embedding protocol, an upgraded light sheet microscope, a comprehensive library of data processing plug-ins, a novel vascular segmentation approach using inherent biological markers and new growth models relying on logarithmic rescaling of time for describing the development of embryonic vascular volume. The presented tools will further open the door for many other long-term imaging studies in fields such as developmental biology or xenograft models in cancer biology.

### Growth of the vasculature

With our multi-sample imaging and analysis platform, we obtained a high-quality data set describing the growth of the vascular volume over time. Descriptive features of the observed time course include a slow growth at the beginning, a maximum volume growth rate at around 26-29 hpf and then a decline of the growth rate resulting in a saturating growth process. To describe this growth, we fit well-established growth models (Fig. S11) that have been used for saturating growth processes, such as Gompertz's, Richards’ and Weibull's models or log-logistic function (Table 1). We found that they did not reproduce the first hours of vascular development well. In searching for an alternative growth model, we found that the best growth models to explain the data all include an effective, logarithmic rescaling of time. Log-normal-like dynamics and log-logistic growth fit best, and are clearly distinguishable from other, non-logarithmic growth models, but not from one another. To our knowledge, this is the first time that such models have been shown to be applicable to developmental processes.

It is not surprising that both the log-normal and the log-logistic growth model describe the data well. For certain ranges of parameters, the shape of their underlying growth rates can be very similar in nature. This is also known from statistics where the analogous log-normal and log-logistic distributions can be applied to right skewed distributions, and an experimenter can choose any one of the two models to describe the data (Dey and Kundu, 2009). Methods to distinguish the two often involve analysing their probability density function (Raqab et al., 2017). In our case, however, only the cumulative function as volume measurement is given. Other methods that rely on the analysis of the tails of the distribution were not applicable, as the observed time of growth was not long enough to unravel effects from the tails of the functions. However, the log-logistic growth model has the advantage that it is more easily interpretable as it has a closed function for the cumulative function contrary to the log-normal growth model. The scaled cumulative log-logistic model thereby is closely related to the logistic equation that is often applied in description of population growth in biology (Verhulst, 1838) or cancer growth (Vaidya and Alexandro, 1982).

One might envision such logarithmic rescaling of time as arising from an effective ‘clock’ that is driven by an exponentially decaying resource. In vasculature development, we speculate that factors such as: (1) nutrient availability; or (2) gene expression could introduce this effect and explain the observed logarithmic rescaling of time. In (1), a limited store of nutrients is deposited by the mother and remains the predominant source of nutrients over the first few days of development. Assuming an exponentially decaying use of nutrients out of this store, if each growth step of the vasculature requires some constant threshold amount of nutrient to proceed, then the growth steps over time become logarithmically rescaled. In (2), gene expression could also introduce similar rescaling effects. Here, feedback loops in gene expression patterns would enforce dependence on the pattern at a previous time point, leading to an exponentially decaying concentration of expression products. Again, if each vascular development step requires a threshold amount of these products, then logarithmically rescaled times would result.

### Growth of vascular subnetworks

The volume measurements of large subnetworks of the vasculature such as the head and the tail vasculature were also well described by scaled cumulative log-logistic and log-normal growth models. This indicates that those large subnetworks were also subject to the same growth constraints as the whole vasculature and explain, in part, the fractal nature of vasculature. However, these constraints might not apply to smaller subnetworks as they can draw from and release resources to neighbouring parts of the network, e.g. by cell migration. Therefore, those smaller subnetworks might show different growth behaviours. Indeed, the caudal vein plexus located in the tail of the vasculature first expanded, then remodelled and, after 73 hpf, decreased in size (Fig. S12), and therefore did not follow a scaled cumulative log-logistic or log-normal growth curve.

### Multi-sample capacity is desired and important

Simultaneous imaging of several samples offers higher efficiency, shorter overall experimental time and therefore lower costs than sequential image acquisition. Furthermore, it enables the study of embryos from the same parents, growing under exactly the same conditions. This is especially important for imaging mutant fish lines. Homozygous mutant fish are often not viable (Kim et al., 2011), and therefore heterozygous fish lines are grown. However, only one quarter of their progeny are homozygous for the mutation of interest and exhibit the phenotype. To observe its establishment, fish are ideally selected before a phenotype is observable. Consequently, multi-sample imaging is essential for studying mutant fish lines with high spatial and temporal resolution.

The ability to image many samples is also important for experiments that are time consuming in preparation and/or rely on the short-term availability of rare, restricted or otherwise difficult to obtain samples. Examples include patient-derived samples, xenotransplantation samples, observations of induced cancer cells or small-scale screens of selected compounds. Our multi-sample platform has already been applied successfully to such a study where the influence of the Rho-kinase inhibitor fasudil was investigated on drug-treated and non-drug-treated fish in the same experiment (Asokan et al., 2017preprint). In future, the platform will provide the required sample capacity to tackle more such projects.

Moreover, with the multi-sample platform, new studies will be possible that aim to understand the variation and robustness of biological structures for which a high sample number is important. Indeed, the vasculature of zebrafish also shows variation (Fig. S13). In future studies, our long-term time-lapse platform will be used to study how such patterns of variation are established, maintained and/or remodelled. We are confident that our segmentation approach will therefore be the starting point for such endeavours. As a demonstration of such a study, we have quantified the symmetry of the intersegmental vessel pairs along the anterior-posterior axis (Fig. S14). Interestingly, the degree of symmetry changes depending on the position along the anterior-posterior axis.

### Integration is crucial

**Fig. 4.**
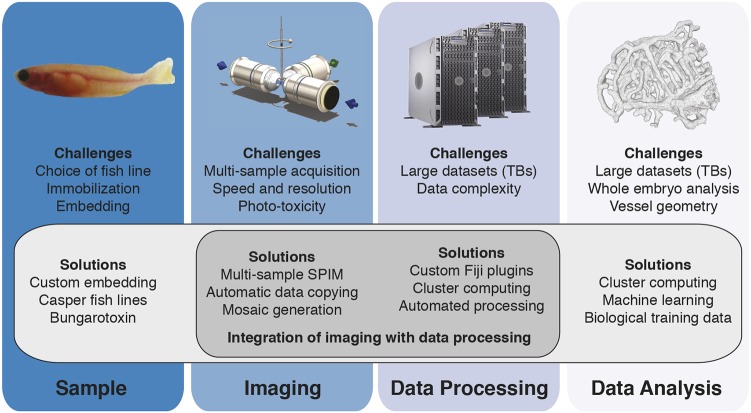
**A dedicated imaging, processing and analysis platform for multi-sample imaging.** Integration of important steps of multi-sample imaging, such as sample preparation, imaging, data processing or data analysis, facilitates the individual steps and enables multi-sample imaging.

The power of our multi-sample imaging platform lies in the integration of all of the important steps of multi-sample imaging – sample preparation, imaging, data processing and data analysis – into a single dedicated pipeline to obtain quantitative data (Fig. 4). For example, the data analysis benefits from the right choice of sample. Only by using the red blood cells as a luminal marker, did we obtain good training data to solve for the segmentation parameters. In a further example, imaging and data processing are interlinked, as the acquisition parameters, here the translational stage positions, are crucial for reliable stitching. As all of our processing and analysis code is freely available, other researchers may adapt parts of the whole pipeline to their dedicated research question. Moreover, our suggested upgrade for multi-sample imaging may be easily incorporated into other custom-built systems, together with the embedding protocol described in detail here. Hence, this toolset will help current imaging strategies move from qualitative descriptions of single observations to quantitative analysis over multiple samples.

## MATERIALS AND METHODS

### Ethics statement

The animal experiments were performed in accordance with the European Union (EU) directive 2011/63/EU as well as the German Animal Welfare Act.

### Zebrafish sample preparation

Zebrafish (*Danio rerio*) adults and embryos were kept at 28.5°C and were handled according to established protocols (Nüsslein-Volhard and Dahm, 2002; Westerfield, 2000). To understand vascular growth in zebrafish, we compared *Tg(fli1a:EGFP)* (Lawson and Weinstein, 2002) and *Tg(kdrl:EGFP)* (Jin et al., 2005)*.* As the marker *Tg(fli1a:EGFP)* was expressed not only in the head vasculature but more broadly in the head (data not shown), we decided to use *Tg(kdrl:EGFP)* and crossed this fish line into a casper background (White et al., 2008) to suppress formation of pigmentation. The *Tg(kdrl:EGFP)* casper fish line was crossed with the line *Tg(GATA1a:dsRed)* (Traver et al., 2003) expressing a fluorescent red blood cell marker. For time-lapse experiments, zebrafish embryos were injected at the one-cell stage with 30 pg of α-bungarotoxin RNA (Swinburne et al., 2015) to ensure immobilization during the time-lapse. A detailed step-by-step protocol for embedding the embryos for imaging is in the supplementary Materials and Methods.

### Growth measurement

Freely swimming zebrafish, single-sample and multiple-sample preparations were set up at 24 hpf. Freely swimming zebrafish (*n*=8), single-sample (*n*=10) and multi-sample (*n*=9) preparations were then imaged using an AVT stingray camera connected to an Olympus SZX16 stereo microscope at 24, 48, 72, 96 and 120 hpf. To calibrate the length measurement, a PYSER-SGI stage micrometer (10 mm/0.1 mm) was imaged together with the zebrafish embryo. Fiji (Schindelin et al., 2012) was used to first calibrate and then measure the length of the zebrafish embryo.

### *In vivo* time-lapse imaging

Long-term time-lapse imaging was performed on a custom-made multidirectional SPIM (mSPIM) setup (Huisken and Stainier, 2007) upgraded to multi-sample capacity (see Results section). The microscope was equipped with two Zeiss 10×/0.2 NA illumination objectives and an UMPlanFL N 10×/0.3 NA Olympus detection objective. Coherent Sapphire 488 nm and 561 nm lasers, each 100 mW maximum power, were used to illuminate the sample. The images were recorded with two Andor DV885 iXon EM-CCD cameras. The embryos were imaged at least every 25 min for up to 4 days starting around 17 hpf.

### Design of the translational stage system

The translational stage system of the microscope required high precision as it positioned the sample and scanned it through the light sheet. Therefore, a precise, translational stage with a longer travel range had to replace the existing vertical translational stage unit (Fig. 1D). We chose the M-404.4PD precision translation stage (Physik Instrumente) as it offered a unidirectional repeatability of 0.5 µm, equal to half a pixel on the camera, and an overall travel range of 100 mm, which was sufficient for imaging several samples. To integrate the larger stage, we designed custom parts to connect the M-404.4PD stage to two M-111.1DG translational stages (Physik Instrumente) for lateral and axial scanning. We further added a solid metal block to stabilize the translational stage system and avoid vibrations caused by translational movements.

### Data copying in-between timepoints

For data transfer, we used the Windows command line executable Robocopy that was started immediately after the acquisition of every 3D stack and copied data until just before a new stack acquisition was started. Robocopy was integrated into Labview, the microscope control software, using their executable interface framework. This ensured robust data transfer, as Robocopy only removed old data once the integrity of the file at the new server location was checked.

### Data processing

Custom-made data processing tools in Fiji (Schindelin et al., 2012) were written to process the data from the microscope automatically. For visualization, the acquired fluorescence images were projected using maximum intensity projections. The resulting projections were stitched and fused with linear blending using custom code adapted from the Fiji stitching plugin by Stephan Preibisch et al. (Preibisch et al., 2009) that relies on phase correlation (Kuglin and Hines, 1975). For successful stitching (Fig. S8), we initialized the stitching with the translational stage positions. As the translational stages were very precise, the stitching was robust and determined globally for all timepoints. The stitching parameters of the maximum intensity projections were also applied to the 3D data to generate one 3D fused stack per timepoint, angle, fish and channel. While the different channels were already aligned optically, we used a manual GUI interface to obtain a precise fine alignment of the different channels using rigid registration. To reduce the amount of data for storage to about half the size, the data was compressed by zipping. Code of the custom-made processing steps is available freely at github.com/DaetwylerStephan/multi_sample_SPIM.

### Segmentation

Segmentation of vasculature is particularly challenging because blood vessels are hollow tubes of widely varying diameters, often in very close proximity to each other. Additionally, endothelial markers often show heterogeneous densities across the vessel walls, e.g. with higher intensities around nuclei. Therefore, classical approaches such as filter-based approaches (Sato et al., 1998) or simple thresholding did not result in good segmentations (data not shown). Consequently, we have designed a novel approach of vessel segmentation (see Results section). The image segmentation software was developed using FunImageJ (Harrington et al., 2018), a Lisp-based interface for ImageJ. Code is freely available at: github.com/kephale/virtualfish-segmentation.

### Visualization of segmentation

To visually check the quality of the segmentation (Fig. 2E), we overlaid the segmented images with the raw signal at selected timepoints. We further visually inspected all segmentations over the whole time-lapse course using maximum intensity projections and by 3D rendering (Fig. 2F,G, Movie 3) the segmentation and the annotations using SciView (available at github.com/scenerygraphics/sciview) or Vaa3D (Peng et al., 2010).

### Quantification of vascular growth

For quantification of the segmentation, we rescaled each segmented 3D stack of a time-lapse series to isotropic resolution and then counted the number of segmented voxels. We quantified only the two opposite angles rotated by ±60° from the sagittal plane. Those two angles provided the best resolution of the whole zebrafish embryo vasculature. To obtain the vascular volume growth curves for one fish, the quantification result of both angles was averaged.

### Annotation of the segmented vasculature

For the annotation, we used the maximum intensity projections of the endothelial signal over time. We first selected manually a region of interest, such as the caudal vein plexus, the head or the tail vasculature (including the caudal vein plexus), at the last timepoint of a time series. To automatically track this region of interest over time, we sequentially determined the region of interest at time t−1 given the region of interest at time t. For this, the boundary of the region of interest was discretized by points. For each point at time t, the corresponding point at time t−1 was determined. Assuming that only small-scale changes were present, the computation for each point was reduced by considering only a subregion of the image at time t−1 (140×140 pixels) as a search image for the template that was a small crop of the image at time t (70×70 pixels concentric around point). Within the search image, smaller images of the size of the template were created and compared against the template using image correlation. The position with the highest similarity was the new place for the boundary point. To increase robustness of the method, the shift vectors [new position at time (t−1)−position at time t] for each point were determined and the median over the seven neighbouring boundary points calculated and assigned as effective shift. Furthermore, if two boundary points were assigned to the same or neighbouring pixel, one of them was removed from the computation. To ensure the quality of the annotation, we visually inspected and curated the annotation.

### Analysis of volume growth

The resulting volume measurements were analysed with the software package R (R Core Team, 2018) using the dyplr library package (Wickham et al., 2018) for data handling. Parameters for different growth models were optimized using non-linear regression of the sum of squared differences between the actual values and the predicted value of the growth model given the parameters. As optimization algorithm, we applied a non-linear least squares approach (nls function in R). In case nls failed, we applied the Levenberg-Marquardt algorithm (nlsLM function in R; Elzhov et al., 2016). Plots were generated using the ggplot library (Wickham, 2016) and the gridExtra package (Auguie, 2017).

## Supplementary Material

Supplementary information
